# Genetic characterization of rat hepatitis E virus (*Rocahepevirus ratti*) in urban brown rats (*Rattus norvegicus*) in Helsinki, Finland

**DOI:** 10.1007/s00705-025-06412-4

**Published:** 2025-09-29

**Authors:** Mert Erdin, Eda Altan, Nina Suomalainen, Hussein Alburkat, Tuomas Aivelo, Tarja Sironen, Teemu Smura

**Affiliations:** 1 Department of Virology, Medicum, Faculty of Medicine, University of Helsinki, Helsinki, Finland; 2 Science Communication and Society, Institute of Biology, University of Leiden, Leiden, The Netherlands; 3 Organismal and Evolutionary Biology Research Program, Faculty of Biological and Environmental Sciences, University of Helsinki, Helsinki, Finland; 4 Department of Veterinary Biosciences, Faculty of Veterinary Medicine, University of Helsinki, Helsinki, Finland

## Abstract

We report complete and partial genome sequences of rat hepatitis E virus (RHEV, *Rocahepevirus ratti*), from archived brown rats captured in Helsinki, Finland. Phylogenetic analysis confirmed the presence of the pathogenic RHEV genotype C1 in the Helsinki region. Finnish strains clustered together with strains from South Korea and Spain. However, the polytomous topology of phylogenetic trees and the large genetic distances between spatially distinct strains suggest that RHEV has remained inadequately sampled on a global scale. Further surveillance of rocahepeviruses is needed to assess their threat to public health and to understand their diversity and evolutionary patterns.

**Supplementary Information:**

The online version contains supplementary material available at 10.1007/s00705-025-06412-4.

Hepatitis E viruses are single-stranded positive-sense RNA viruses belonging to the family *Hepeviridae*, which is further divided into two subfamilies: *Orthohepevirinae* and *Parahepevirinae* [1]. *Rocahepevirus* is one of the four genera in the subfamily *Orthohepevirinae*, and this genus is known to include genotypes that are pathogenic to humans [1]. Initially, rocahepeviruses were discovered in rodents, particularly in rats [2], but they have recently been found to circulate also in mice, voles, and even carnivores such as ferrets [3]. Currently, the genus *Rocahepevirus* is divided into two species: *Rocahepevirus ratti* (rat hepatitis E virus, RHEV) and *Rocahepevirus eothenomi* (vole hepatitis E virus, VHEV). VHEV has been reported in voles (family Cricetidae, order Rodentia) [4, 5], whereas RHEV has been detected in rodents, ferrets (ferret-borne RHEV is also known as ferret hepatitis E virus, FrHEV), and foxes (order Carnivora), as well as humans [2–11]. Currently, there are two officially classified RHEV genotypes according to the International Committee on Taxonomy of Viruses (ICTV): RHEV-C1 in rats and RHEV-C2 in mustelids. In addition, a putative genotype infecting field mice has been proposed [12].

While human hepatitis E virus (HEV; subfamily *Orthohepevirinae*, species *Paslahepevirus balayani*) is one of the most common causes of acute hepatitis [13, 14], rocahepeviruses have garnered attention due to their zoonotic potential. In addition to acute hepatitis, HEV is associated with severe hepatitis in women during pregnancy [15]. Some adverse outcomes during pregnancy, such as fulminant liver failure, premature delivery, postpartum hemorrhage, and low birth weight, may be observed and pose a threat to both mothers and infants [15]. Thus, understanding the diversity of HEV and RHEV will help to improve diagnostic methods for early detection and consequently enhance our capability to prevent these adverse outcomes. Genotype RHEV-C1 has been reported to cause mild self-limiting liver dysfunction, but it also has the potential to cause persistent infections, especially in immunocompromised patients [9, 11, 16]. Its ability to cause infections in humans may add to the global human hepatitis E burden and makes this virus a public health concern. Human case reports of RHEV-C1 from Hong Kong, Spain, and France and one reported infection in a patient traveling from Africa to Canada have provided insight into the global distribution and high divergence of these viruses [8–11]. The incidence of RHEV-C1 infections seems to be higher in Europe and Asia than on the other continents [17], but more studies are needed to confirm this.

Rats are distributed globally, and therefore, human pathogens carried by these animals are also widespread [18]. Rats co-inhabit areas with humans, increasing the likelihood of direct or indirect contact between the two. This highlights the importance of pathogen surveillance in rats. The genus *Rocahepevirus* seems to have high genetic diversity with varying zoonotic potential, as it includes some genotypes that are pathogenic to humans and others that are not. Hence, genetic surveillance of these viruses is of immense importance. Here, we report complete genome sequences and phylogenetic analysis of RHEV from brown rats captured in Helsinki, Finland.

RHEV was detected recently in brown rats (*Rattus norvegicus*) in Helsinki, Finland, and a detailed description of rodent capture, identification, and RHEV screening was provided in a previous report [18]. In that study, the rats were collected by pest management professionals and identified morphologically. A total of 285 rat livers were used for total RNA extraction followed by hepevirus-specific PCR screening, resulting in four RHEV-RNA-positive samples. Here, we subjected these archived RHEV RNA-positive samples to metatranscriptomic sequencing.

We used an NEBNext rRNA Depletion Kit v2 and an Ultra II RNA Library Prep Kit for construction of a next-generation sequencing (NGS) library, which was sequenced using an Illumina NovaSeq 6000 system. The raw data were then quality-filtered and assembled *de-novo*, and the resulting contigs were annotated using Lazypipe software [19]. Two partial and two complete RHEV genome sequences were obtained from a total of four samples. The sequence read and mapping statistics are shown in Supplementary Table S1.

Multiple sequence datasets were used for phylogenetic analysis. The first two datasets consisted of the complete open reading frame 1 (ORF1) and ORF2 sequences of members of the subfamily *Orthohepevirinae* available in the GenBank database. These ORFs encode the non-structural proteins and the capsid protein, respectively. The sequences in each dataset were aligned using MAFFT, and maximum-likelihood (ML) trees were constructed using IQ-TREE2 [20]. ModelFinder [21], implemented in IQ-TREE2, was used to find the best-fitting model for tree construction. This analysis showed the phylogenetic placement of the Finnish strains within the genus *Rocahepevirus*. After this initial characterization, we analyzed a dataset consisting of all of the complete genome sequences of RHEV from brown rats (n = 12). Because of the small number of complete sequences available for RHEV from brown rats, we also included partial sequences of both ORF1 and ORF2 within the complete genome dataset for a total of 221 sequences. The partial sequences from ORF1 and ORF2 ranged in size from 151 to > 6600 nucleotides. These sequences were aligned, and ML trees were constructed as described above. Based on the clustering pattern, we subsampled the sequences to focus on the characterization of the Finnish sequences. Because of the inconsistent length of the sequences in the dataset, we also subsampled the complete sequences and calculated the p-distances, using the ape package in R. The phylogenetic trees were visualized using iTOL or the ggtree package in R.

In a previous study [18], we identified four rat liver samples, one from 2020, two from 2022, and one from 2023, that were found to be positive for HEV using a molecular screening test [18]. These samples were from three different locations: the Vantaa waste incineration plant, from which one positive sample was collected in 2020, the Konala neighborhood, from which two positive samples were collected in 2022, and the Ruskeasuo community garden, from which one positive sample was collected in 2023. The positive samples were sequenced, resulting as two complete RHEV genome sequences and two partial sequences (accession numbers PP839290-93). While one of the partial sequences was a nearly complete genome sequence with 96.5% coverage in comparison to the complete RHEV genome, the other one had only 8% coverage and corresponded to the non-structural protein coding region of the RHEV genome. The nucleotide sequence identity values between the sequences were high (99.3%). In addition to RHEV, two of the samples contained partial genome sequences that were related to Fesa-like virus V5B (MG571885), with 86% nucleotide and 94.4% amino acid identity. Fesa-like virus V5B is an unclassified member of the order *Picornavirales* that was detected in fecal samples collected from Amerindian children living in an isolated Amazonian village in Venezuela [22]. Further studies are needed to assess the prevalence of this virus in rats and to confirm the complete genome sequences. In addition, some sequence reads mapping to members of the family *Partitiviridae* were also detected.

Maximum-likelihood phylogenetic trees based on ORF1 or ORF2 sequences from members of the subfamily *Orthohepevirinae* showed that the RHEV genomes from Finland formed a subcluster together with sequences from Germany, Hungary, the Netherlands, the USA, China, and Indonesia, suggesting a global distribution of this subcluster (Fig. 1). There were only minor differences in the topologies between the ORF1 and ORF2 trees.

**Fig. 1:**
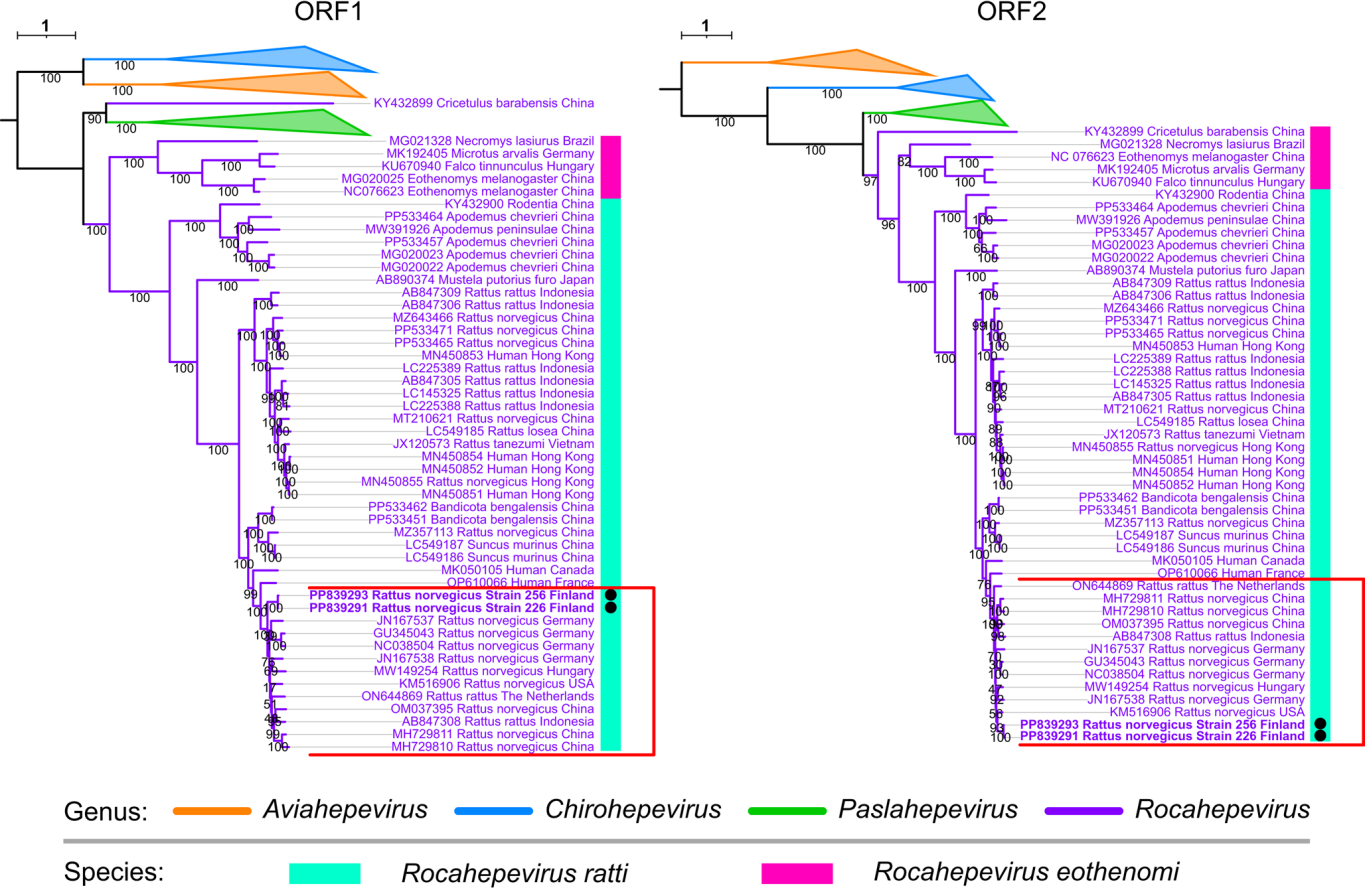
Maximum-likelihood phylogenetic trees based on the ORF1 and ORF2 genes of members of the subfamily *Orthohepevirinae* for genus-level placement of the Finnish strains. The ORF1 tree was constructed using the model GTR + F + I + R5, which was identified as the best-fitting model by ModelFinder, and the ORF2 tree was constructed using TIM2 + F + I + R5 as the best-fitting model. For both trees, 1000 ultrafast bootstrap replicates were performed. The Finnish strains from this study are indicated by black circles, and the subcluster including the Finnish sequences is indicated by a red rectangle

Beacuse the total number of complete genome sequences for RHEV from brown rats (clade RHEV-C1) was small (Supplementary Fig. S1), we combined the complete and partial sequence datasets to better capture the diversity of RHEV-C1. The initial dataset, which included all of the RHEV-C1 sequences, contained 2198 distinct patterns with 1856 parsimony-informative, 607 singleton, and 4562 constant sites. The resulting maximum-likelihood tree exhibited two major clades (Fig. 2). The clade including the Finnish strains was then subsampled and found to contain 2096 distinct patterns with 1801 parsimony-informative, 568 singleton, and 4656 constant sites. In the resulting ML tree, the Finnish strains formed a distinct cluster that shared an ancestral node with complete genome sequences from South Korea and partial sequences from Spain. Notably, the phylogenetic tree exhibited long branch lengths between the tips and internal nodes, suggesting that the full extent of RHEV genetic diversity may not be represented in the tree. Concordantly, relatively high pairwise p-distances indicated a high degree of divergence among the complete sequences (Supplementary Fig. S2)

**Fig. 2:**
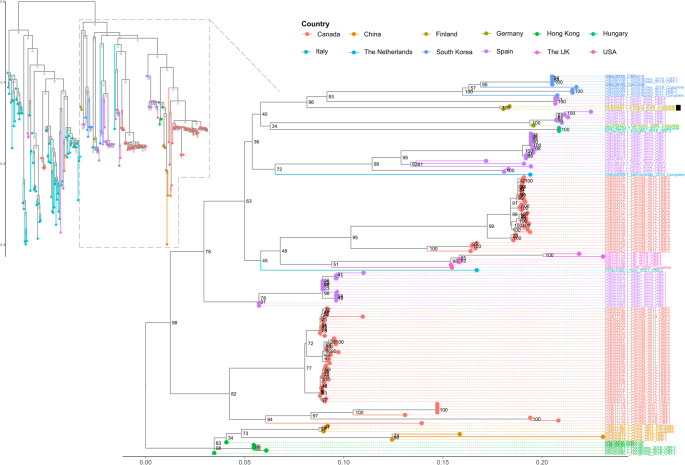
ML trees based on combined datasets including both complete genome sequences and partial ORF1 and ORF2 sequences of RHEV isolates. The partial sequence lengths varied between 151 and > 6600 nucleotides (Supplementary Fig. S1). The ML tree at the left illustrates the overall phylogenetic relationship of isolates of the RHEV-C1 genotype (RHEV strains from brown rats). The tree was constructed using GTR + F + R3 as the best-fitting model with 1000 ultrafast bootstrap replicates. The ML tree on the right, based on the clade containing the Finnish strains, showed high bootstrap values (as percentages) at the internal nodes closer to tips, while the basal nodes had low bootstrap support. This tree was constructed using GTR + F + I + G4 as the best-fitting model, with 1000 ultrafast bootstrap replicates. The Finnish strains are indicated by black rectangles

In addition, low bootstrap values in the basal nodes of the tree indicated poor resolution of deep evolutionary relationships. This suggests that a large number of divergent strains have not yet been identified, especially in Eurasian countries.

In this study, we sequenced the complete genomes of RHEV-C1 genotype isolates from previously identified archived brown rat samples from Helsinki, Finland. The zoonotic potential of brown-rat-associated RHEV raises concerns for human health. Most of the human cases reported to date have been caused by members of genotype C1, to which the Finnish strains also belong. The capability of this genotype to cross host species barriers and the distribution of its main hosts, brown rats, in the areas where humans might come into close contact, makes this genotype a potential public-health threat and highlights the importance of continuous surveillance. It should also be noted that RHEV infections may be under-diagnosed due to the mild and self-limiting disease it typically causes. Further research and more genomic sequencing are needed to understand the spatiotemporal patterns and evolution of RHEV.

## Electronic Supplementary Material

Below is the link to the electronic supplementary material

Supplementary Material 1

## Data Availability

The NCBI GenBank accession numbers for the sequences obtained in this study are PP839290-93.
