# Supplementary material for: Genetic characterization of rat hepatitis E virus (Rocahepevirus ratti) in urban brown rats (Rattus norvegicus) in Helsinki, Finland
Source: Arch Virol. 2025 Sep 29;170(10):215. doi: 10.1007/s00705-025-06412-4 (PMC12479675; doi:10.1007/s00705-025-06412-4)
Supplement: Supplementary file 1 — Supplementary Material 1 [file 705_2025_6412_MOESM1_ESM.docx]

**Supplementary Material**

Supplementary Table 1. The table illustrates the metatranscriptomic sequencing outputs per RHEV positive sample.

| Sample ID | Total reads | Reads passed filter | Reads mapping to RHEV | Mean  coverage | Median coverage |
| --- | --- | --- | --- | --- | --- |
| 221 | 114013014 | 100589656 | 176 | 9.7 | 6 |
| 226 | 21123258 | 15808910 | 265645 | 8615 | 4981 |
| 227 | 4079574 | 2781446 | 340 | 41.9 | 21 |
| 256 | 6378816 | 5797638 | 1969 | 4.04 | 3 |


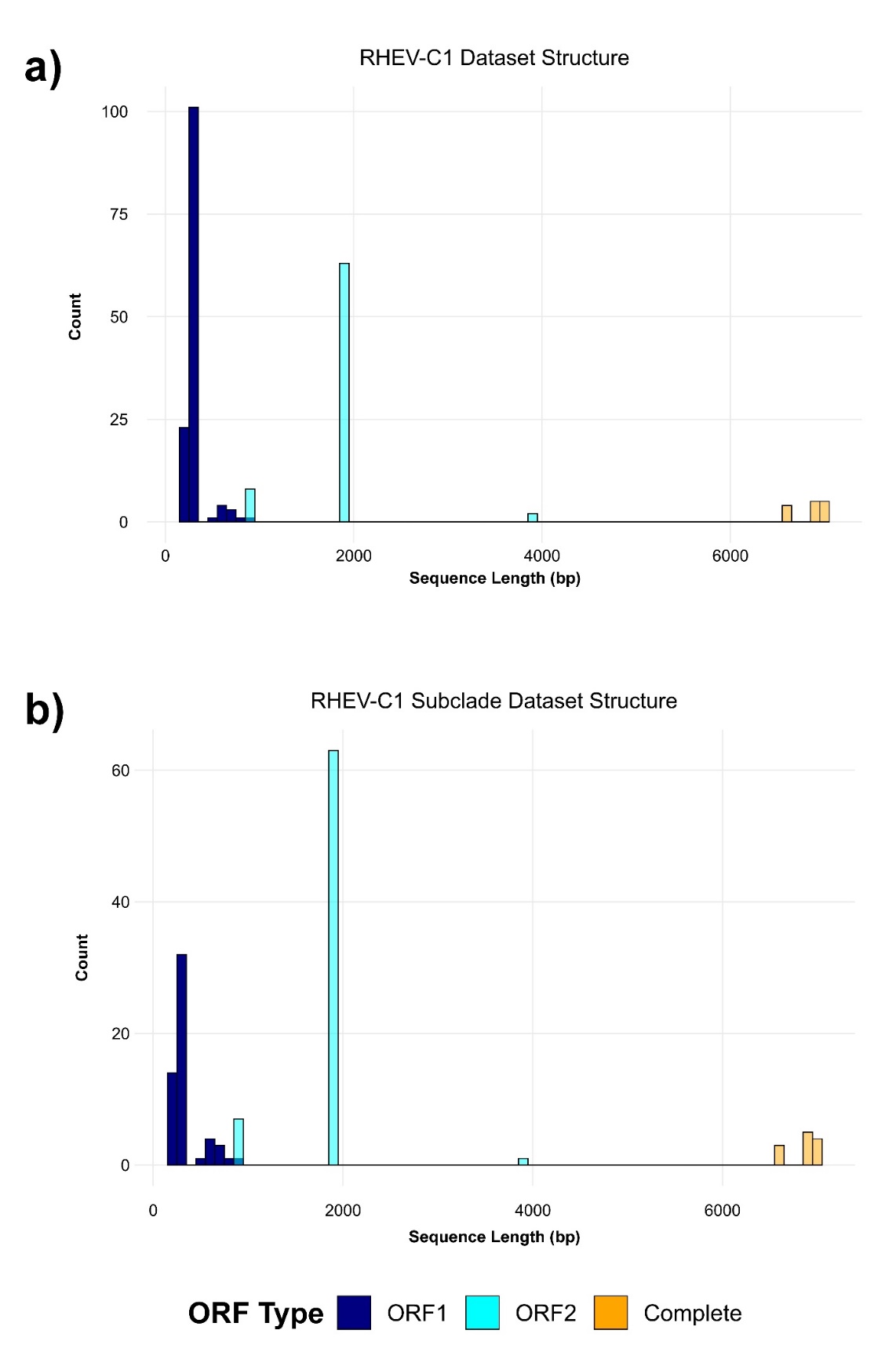


Supplementary Figure S1: The structure of datasets in the sense of sequence lengths. A) The panel represents the entire dataset used for RHEV from brown rats. B) The plot shows the total counts versus sequence positions for the subset where Finnish strains clustered in.


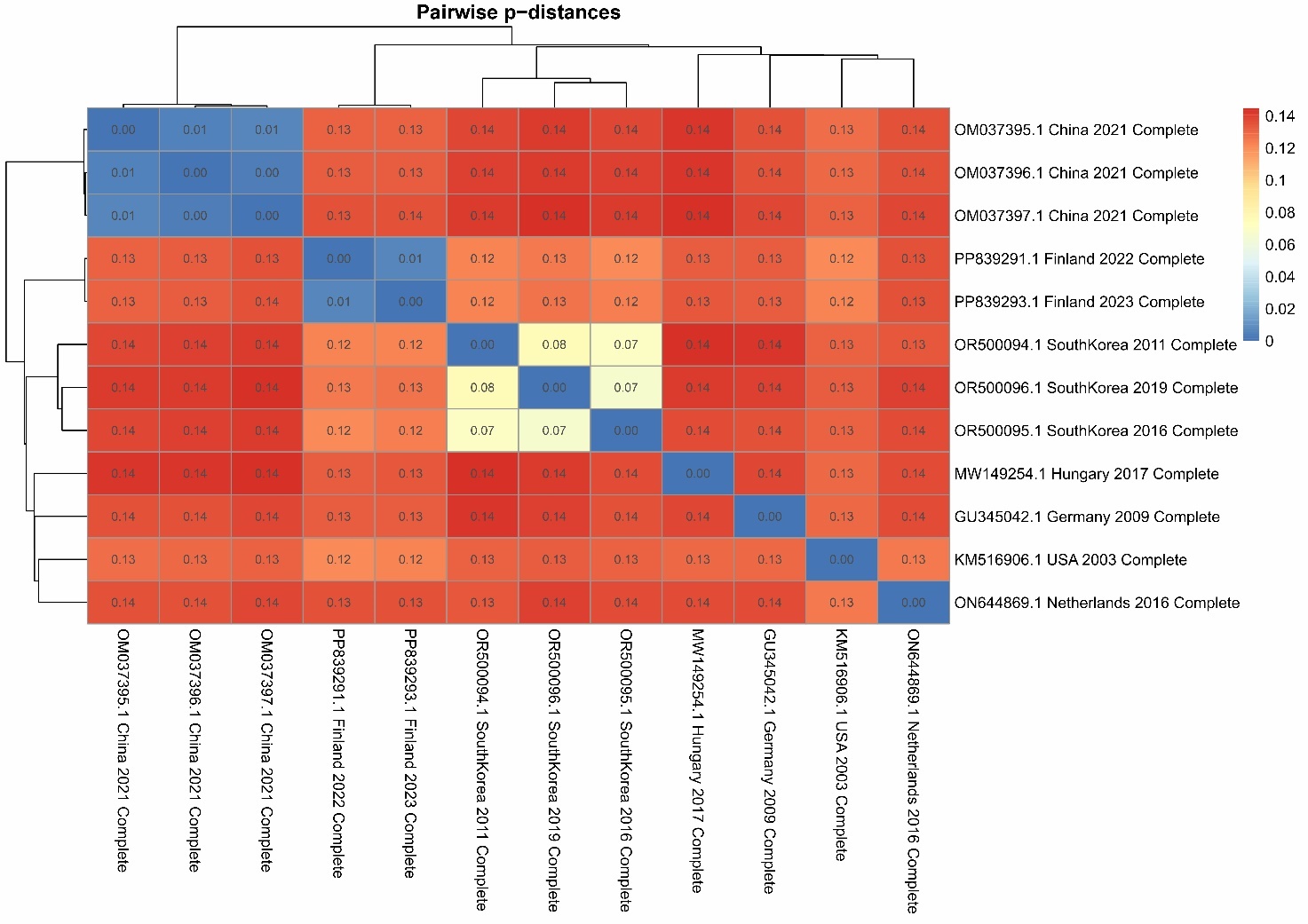


Supplementary Figure S2: The heatmap illustrates the pairwise p-distance between complete sequences from brown rats.
